# MultiFLEXX - The new multi-analyzer at the cold triple-axis spectrometer FLEXX

**DOI:** 10.1038/s41598-017-14046-z

**Published:** 2017-10-20

**Authors:** Felix Groitl, Rasmus Toft-Petersen, Diana Lucia Quintero-Castro, Siqin Meng, Zhilun Lu, Zita Huesges, Manh Duc Le, Svyatoslav Alimov, Thomas Wilpert, Klaus Kiefer, Sebastian Gerischer, Alexandre Bertin, Klaus Habicht

**Affiliations:** 1 Laboratory for Quantum Magnetism, École Polytechnique Fédérale de Lausanne, 1015 Lausanne, Switzerland; 2 Laboratory for Neutron Scattering and Imaging, Paul Scherrer Institut, 5232 Villigen, Switzerland; 3 Helmholtz-Zentrum Berlin für Materialien und Energie, D-14109 Berlin, Germany; 4 Department of Physics, Technical University of Denmark, DK-2880 Kgs. Lyngby, Denmark; 5 Institut für Festkörperphysik, TU Dresden, D-01062 Dresden, Germany; 6 China Institute of Atomic Energy, Beijing, 102413 China; 7 ISIS Neutron and Muon Source, Rutherford Appleton Laboratory, Chilton, Didcot OX11 0QX UK; 8 University of Stavanger, 4036 Stavanger, Norway

## Abstract

The first experimental characterization of a multiple energy analysis wide angle backend for a cold triple-axis spectrometer is reported. The multi-analyzer module MultiFLEXX employs 155 detection channels which simultaneously probe an extensive range in wavevector and energy transfer. Successful mapping of magnetic excitations in MnF_2_ and Ho demonstrate order of magnitude gains in data collection efficiency using this novel type backend. MultiFLEXX is competitive to standard triple-axis spectroscopy in terms of energy resolution and signal-to-noise ratio. A minority of the detector channels is affected by spurious signals inherent to this multiplexing concept. The characteristic signature of these spurious signals easily allows for their discrimination. The instrument concept focuses on detection efficiency in the horizontal scattering plane which makes it an ideal technique for fast mapping and parametric studies including extreme sample environment.

## Introduction

Inelastic neutron scattering (INS) is currently the only technique available to probe low-energy collective excitations in materials with sub-meV resolution. For a broad variety of phenomena in condensed matter physics, parametric studies of low-energy collective dynamics are of particular interest. Quantum magnets are good examples, where a control parameter, such as an applied magnetic field or pressure, induces a quantum phase transition at a critical point where the nature of the collective magnetic dynamics is altered^1–4^. Another example are disordered magnetic systems, which show dynamic correlations covering a large portion of (${\bf{Q}},\omega$)-space, with wavevector transfer **Q** and energy transfer $\hslash \omega$. In investigating such broad features, efficient mapping of the accessible $({\bf{Q}}{\boldsymbol{,}}\omega {\boldsymbol{)}}$-space has priority over high resolution^5–7^.

Mapping experiments, which only depend on temperature and give an overview of the dynamic structure factor S $({\bf{Q}},\omega )$, are usually done on a time-of-flight (ToF) spectrometer. The particular advantage of ToF spectrometers is the large coverage of $({\bf{Q}},\omega )$-space. This is achieved by recording a wide range of scattered energy transfers in combination with a large solid angle covered by position sensitive detectors (PSDs). The downside of the technique is its relatively low incoming neutron flux at continuous neutron sources. Due to the relatively low flux, for sufficient statistics long data acquisition times are required limiting the number of data points as a function of the external parameter. Parametric studies often include the use of sample environment (SE), such as high field cryo-magnets and/or pressure cells. Such SE significantly decreases the performance of ToF spectrometers, since a large fraction of the covered solid angle is blocked and the background is increased. Therefore, such experiments are best carried out at triple-axis spectrometers (TAS). Here, a higher incident neutron flux is focused in the horizontal scattering plane and one single point in $({\bf{Q}},\omega )$-space is probed per scan point. In the past TAS instruments have been improved by increasing the incoming neutron flux by optimizing neutron guides and employing focusing monochromator and analyzer geometries. This resulted in relatively short counting times and made TAS well suited for parametric studies in limited volumes of $({\bf{Q}},\omega )$-space. However, for parametric mapping studies still long acquisition times are required.

A very promising way to increase the data collection efficiency of TAS instruments for parametric studies is the so-called multiplexing technique, i.e., employing several $({\bf{Q}},{E}_{f})$-channels to cover a large range of $({\bf{Q}},\omega )$ in the horizontal scattering plane. Here ${E}_{f}$ defines the final energy of the scattered neutrons analyzed in the corresponding channel. Retaining the initial horizontal scattering geometry of the secondary spectrometer results in spatial constraints and leads to rather complicated technical solutions, such as the RITA spectrometer^8,9^, at Risø, Denmark, where the efficiency is increased by “local” multiplexing without covering a large solid angle. Similar realizations of this concept are RITA-II^10,11^, at SINQ, Switzerland, UFO^12,13^, and IMPS^14^ at ILL, France, and the multi-analyzer system at PUMA^15^ at FRM II, Germany. For these type of instruments spatial restrictions limit the number of possible $({{\bf{k}}}_{f},{E}_{f})$-channels, where ${{\bf{k}}}_{f}$ is the final wavevector of the scattered neutron and the overall gain is simply limited by the analyzer area. The efficiency can be further increased by covering a larger $({\bf{Q}},\omega )$ volume using wide-angle multiplexing. This is realized with MADbox^16,17^, at ILL, France, and MACS^18^ at NIST, USA. Another way to increase angular coverage is to leave the horizontal scattering plane and use a vertically scattering backend. Realizations are the former Flat-Cone^19^ installed at the E2 diffractometer at BER II, Berlin, and the implemented FlatCone at the ILL^20^. However, despite their large angular coverage, these spectrometers record only one final energy ${E}_{f}$ per $2\theta$-channel. The next step in improvement is the use of successive arrangements of vertically scattering analyzer crystals accepting multiple final energies ${E}_{f}$ in a single $2\theta$-channel. This is known as the Continuous Angle Multiple Energy Analysis (CAMEA) backend^21,22^. The concept is possible due to the transparency of highly oriented pyrolytic graphite (HOPG) at large wavelengths^23^. A CAMEA type backend is under construction for the cold neutron TAS (cTAS) RITA-II at the PSI^22^ and in the design phase for the PANDA cTAS^24–26^, at the MLZ facility^27^. Furthermore, this type of backend will be employed in the high performance indirect ToF spectrometer BIFROST to be built at the ESS^21^. As a BIFROST prototype the CAMEA concept has already been successfully tested on a ToF frontend^28^. This led to the newly developed prismatic analyzer concept^29^. However, none of these CAMEA backends has so far been realized with a standard cTAS frontend.

Here, we report on the new multiplexing backend MultiFLEXX which has been designed for the cTAS FLEXX at the BER II neutron source, HZB^30,31^. Preliminary measurements on a two-channel prototype version of MultiFLEXX on the cTAS PANDA at the MLZ have been reported earlier^32^. Here, the performance of MultiFLEXX is discussed in detail. The article is structured as follows. First the design concept is introduced, which addressed two challenges: simplicity in operation and minimization of background. Secondly, characterization measurements focusing on energy resolution, detector efficiency and data normalization are presented. The capabilities of the instrument are demonstrated by mapping out dispersions in MnF_2_ and Ho. The experimental section concludes with a detailed analysis of spurious signals and background.

## Instrument Design

### FLEXX

The cTAS FLEXX is located at a guide-end position in an area of the guide hall with intrinsically low background. The primary spectrometer employs new $m=3$ guides ($60\times 125\,$ mm^2^) including a converging elliptical section with a supermirror coating gradually increasing from $m=3$ to $m=5$ to focus neutrons onto a vertical virtual source^30,31^. A double focusing monochromator (PG002, 300 mm width and $140\,$mm height) images the neutrons subsequently onto the sample position. This increases the overall flux on the sample at the expense of a coarser **Q**-resolution. The energy resolution remains unaffected by satisfying Rowland geometry focusing conditions^33^. A velocity selector placed far upstream in the primary beam path removes higher-order scattering from the monochromator and reduces the background which is vital for inelastic neutron scattering experiments. However, second-order scattering from the analyzers occurs at energies 10, 12, 14, 16 and 18 meV. Consequently, for incident neutron energies above 10 meV, elastic incoherent scattering from the sample gives rise to spurious signals due to second-order scattering from the analyzer crystals. This can be straightforwardly removed by constructing a wide-angle beryllium filter with a cut-off energy at $E > 5$meV.

### Design of MultiFLEXX

**Figure 1:**
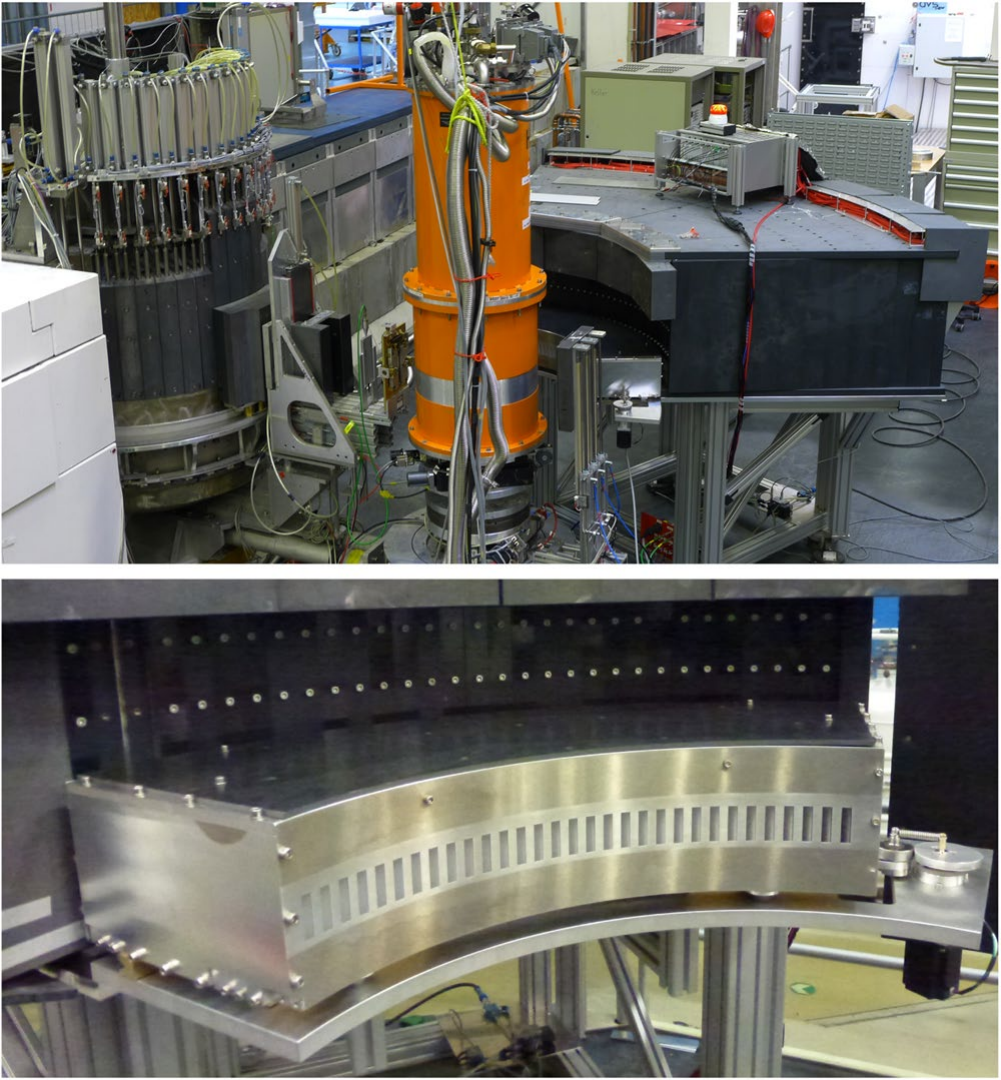
Top: The cold neutron TAS FLEXX with the multi-analyzer module MultiFLEXX installed. Bottom: The oscillating radial collimator mounted in front of the MultiFLEXX channels.

The MultiFLEXX backend was designed to remain optional, only to be used for overview studies when called for on an experiment-to-experiment basis. Hence the module was designed for both robustness and simplicity in operation. The latter was achieved by minimizing the amount of variable parameters: the incident energy ${E}_{i}$, the sample rotation angle $A3$ and the scattering angle $A4$ determining the angular position of the whole module with respect to the incident beam and thus the angular range covered by MultiFLEXX. Thus, a minimum calibration effort is required and the module is easily installed during experiments. The resulting MultiFLEXX backend module is shown in Fig. 1. An interchange between the standard TAS analyzer-detector module and the multi-analyzer module MultiFLEXX takes as little as 20 minutes. The 155 analyzer channels of MultiFLEXX are distributed over 31 angular segments ($2\theta$-channels) spanning a 77.5° scattering angle with an angular separation between channels of 2.5°.

**Figure 2:**
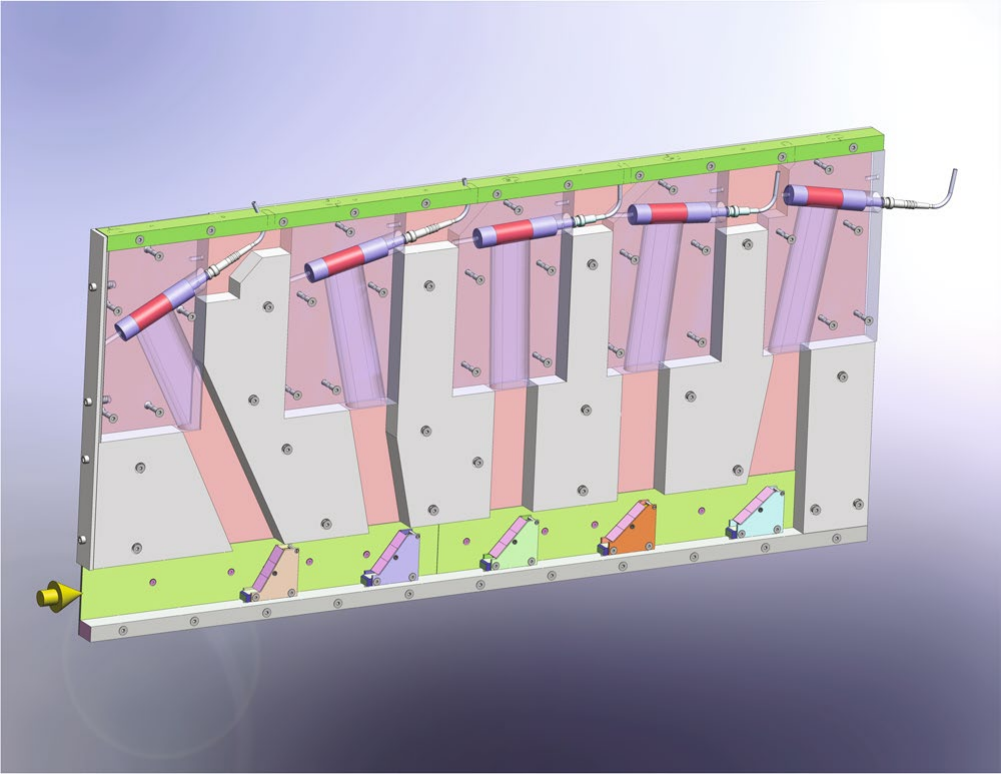
Layout of a single angular segment (side view), the distance between the analyzers at the bottom (purple, mounted on holders) and the detectors at the top (illuminated length marked in red) is 400 mm. Neutrons scattered by the sample enter the segment from the left as indicated by the arrow. The distances of the analyzers to the sample are 1050 mm, 1220 mm, 1387 mm, 1552 mm and 1732 mm, respectively. This corresponds to vertical angular acceptance angles of ±1.40°, ±1.10°, ±0.89°, ±0.75° and ±0.63° for an ideal point-like sample, while the horizontal acceptance angles are ±0.55°, ±0.47°, ±0.41°, ±0.37° and ±0.33°, respectively.

Figure 2 shows the design of a single $2\theta$-channel, which is built as a cassette - hosting analyzers, detectors and shielding - to be slid into the main shielding module. Each channel contains five fixed analyzers (energy channels) accepting final energies ${E}_{f}$ of 2.5, $3.0$, $3.5$, $4.0$ and $4.5\,$meV, thus covering an energy-transfer interval of $2\,$meV. The absolute energy transfer-range in an experiment is then determined by the incident energy ${E}_{i}$, fully adjustable within the range of the standard FLEXX ($1.9\,$meV < *E*_*i*_ < $20\,$meV). MultiFLEXX has been designed and optimized to host the HZB sample environment suite. For sample environment such as high field magnets or pressure cells, the vertical out-of-plane scattering is limited. Thus, the out-of-plane angular coverage of the analyzers was adapted accordingly (between ±1.4° and ±0.6° depending on the analyzer-sample distance).

**Figure 3:**
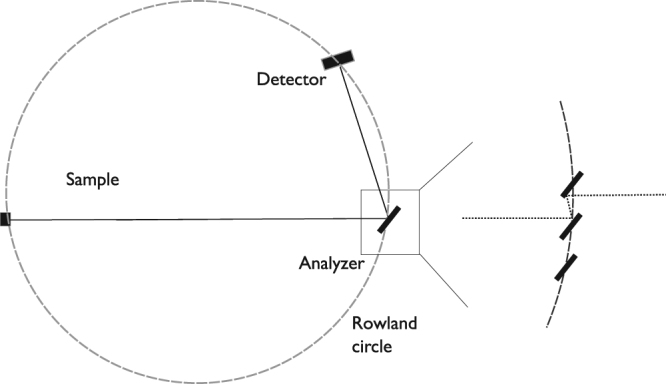
Sketch of the asymmetric Rowland geometry for the MultiFLEXX design with a short analyzer-detector distance. Placing the three analyzer crystals on the Rowland circle would result in significant double scattering, limiting the channel performance. This double scattering effect would be present in all 5 analyzer arrays, where the Rowland geometry is highly asymmetric in all cases. Thus a different geometry for the analyzers was used in the design of MultiFLEXX (see text).

All analyzers are composed of 3 vertically stacked plate-like $20\times 20\times 2$ mm^3^ HOPG crystals (mosaicity of 0.4° ± 0.1° measured with X-rays, purchased from Optigraph GmbH) mounted in a fixed focusing geometry. The analyzer area is therefore constant. However, the vertical angular coverage remains a function of the sample-analyzer distance and the analyzer take-off angle. In order to avoid additional background the aluminum holders of the HOPG crystals are designed such, that the amount of scattering material in the beam is minimized. To minimize both cost and weight of the entire MultiFLEXX module, a small analyzer-detector distance of 40 cm was used. This allows for the use of small and affordable ^3^He tube-detectors (radius of 12.5 mm, active length of 50 mm, purchase from GE Reuter-Stokes) for each analyzer which cover a significant solid angle. The corresponding asymmetric Rowland geometry is shown in Fig. 3. For optimal energy resolution, the spatial position of the analyzer crystals should ideally be on the Rowland circle. However, as evident from Fig. 3, the short analyzer-detector distance prevents such an analyzer arrangement as multiple scattering will significantly reduce performance. Consequently the analyzer crystals were arranged as a plate-like extension of each other. The optimal curvature of the analyzers was simulated using the McStas ray-tracing package^34,35^. Due to the large solid angle covered by the detector, the analyzer curvatures are rather small. The sample-analyzer distances, curvatures and simulated elastic linewidths are given in Table 1 ^36^. As evident in Table 1, there is a discrepancy between the simulated and measured linewidths, most significant at the lower analyzer energies. This is due to the fact that the vertical divergence of the incident beam is larger than the simulated one. This impacts the energy resolution of the vertically scattering analyzers. Due to the distance collimation of the analyzers furthest from the sample, this effect is largest for the low-energy analyzers closest to the sample.

**Table 1:** The specifications of the analyzer parameters used for MultiFLEXX. All analyzers are composed of 3 HOPG crystals of 20 × 20 mm^2^ cross section.

*Channel*	2.5 meV	3 meV	3.5 meV	4 meV	4.5 meV
${D}_{sample}$ [mm]	1050	1220	1387	1552	1732
${\rm{\rho }}$ [1 /m]	0.76	0.6	0.5	0.41	0.37
${\rm{\Delta }}{E}_{sim}^{FWHM}$ [$\mu$ eV]	53	78	103	130	157
${\rm{\Delta }}{E}_{meas}^{FWHM}$ [$\mu$ eV]	89	106	123	140	164
take-off angle $2{\theta }_{A}$ [deg.]	116.98	102.20	92.20	84.74	78.90
${k}_{f}$ [${{\rm{\AA }}}^{-1}$]	1.1	1.2	1.3	1.39	1.47
${\rm{\Omega }}$ [a.u.]	1	0.68	0.48	0.36	0.27
${V}_{res}$ [a.u.]	1	1.70	2.57	3.59	4.71
Normalization factor	1	1.16	1.23	1.30	1.27

The elastic linewidths ${\rm{\Delta }}{E}_{sim}^{FWHM}$ were simulated using McStas, and the tabulated measured elastic linewidths ${\rm{\Delta }}{E}_{meas}^{FWHM}$ are averages over all analyzers with the same ${E}_{f}$. The curvature of the analyzer segments is given by ρ.  The covered solid angle ${\rm{\Omega }}$ has been calculated from the sample-analyzer distance ${D}_{sample}$ and the take-off angle $2{\theta }_{A}$. The resolution volume ${V}_{res}$ is calculated directly from the take-off angle and the final wavevector ${k}_{f}$. Both variables are normalized to the values for the ${E}_{f}=2.5\,$meV analyzer.

Taking the analyzer distances and dimensions into account the horizontal angular coverages of the analyzer segments with energies from 2.5 to 4.5 meV are 1.09°, 0.94°, 0.83°, 0.74° and 0.66°, respectively. With an angular separation of 2.5° between each segment the corresponding dark angles are 1.41°, 1.56°, 1.67°, 1.76° and 1.84°, respectively. This allows a coverage of the entire $2\theta$-range of MultiFLEXX with a sufficient sampling density by two scans which are separated by ${\rm{\Delta }}2\theta ={1.25}^{\circ }$. Given the large incident horizontal divergence defined by the monochromator which dominates the horizontal wavevector resolution a smaller step size in $2\theta$ is not required.

The shielding of the backend is constructed as follows: Each $2\theta$-channel is built as an aluminum cassette to be slid in and out of designated positions in the outer borated polyethylene (BPE) shielding. The cassette interior is lined with a thin Cd-sheet (1 mm) and each analyzer detector channel is thoroughly shielded with BPE to prevent cross talk between channels and avoid a direct view between detectors and neighboring HOPG analyzers. The outermost detectors are additionally shielded with thin Cd-sheets. In order to reduce the background arising from sample environment, MultiFLEXX employs an oscillating radial collimator with inner and outer radii of 473 mm and 673 mm, respectively. The radial collimator consists of Gd coated glass plates of 0.5 mm thickness with an opening angle of 1.25°. The oscillating collimator is mounted onto a translation stage which allows to cover the whole angular range of MultiFLEXX avoiding dark angles. It is designed such, that it fits with the existing sample environment suite available at FLEXX. A velocity selector employed at FLEXX^30^ already suppresses higher order neutrons, which would give rise to additional background. Thus, the implementation of an additional beryllium filter is dispensed.

The detectors of MultiFLEXX are optimized for the different final energies of the backend. The ^3^He fill pressure ($3.5$, $3.8$, $4.1$, $4.4$ and $4.7\,$bar) of the detector tubes of the different energy channels is adapted to provide the same detection efficiency for the different final wavelengths. The analog detector signals are analyzed with remotely controlled readout modules MSTD-16 (Mesytec GmbH), which allows for an individual optimization of the gain factor for each detector channel. Groups of $16$ detectors have a common discrimination threshold. This allows to work with only two different nominal high voltage settings for the whole detector arrangement. The data of the $10$ readout modules are processed by two data collection modules MCPD-8 (Mesytec GmbH) which allow for event mode recording including time stamping. The necessary readout modules including the built-in pre-amplifiers are directly mounted on top of the multiplexing backend. This optimizes signal amplitudes and reduces the number of cables for data transfer and high voltage to a minimum. These cables can easily follow the instrument movement during a scan.

### Normalization

Normalizing intensities becomes a challenge in multiplexing backends like MultiFLEXX. In addition to the normalization of the individual count rates, correcting for stochastic differences in reflectivity and detector efficiency, it is also necessary to take the systematic differences in ${k}_{f}$ and covered solid angle ${\rm{\Omega }}$ into account. The collected intensity for a given ${k}_{f}$ is proportional to the solid angle covered by the analyzer in question around the sample. Due to varying take-off angles $2{\theta }_{A}$ and sample-analyzer distances in MultiFLEXX, the solid angle is reduced with distance from the sample. This effect is balanced out by the volume of the instrument resolution function ${V}_{res}$ which increases with increasing ${k}_{f}$, resulting in a larger part of the dispersion surface being measured. Correspondingly, the systematic variation in intensity can be described by the expression

$$I\propto {\rm{\Omega }}{V}_{res}={\rm{\Omega }}\frac{{k}_{f}^{3}}{tan({\theta }_{A})}\mathrm{.}$$

The relative values of these constants for the analyzers of the MultiFLEXX are given in Table 1, to be used for normalization of integrated intensities.

## Performance

### Energy resolution

**Figure 4:**
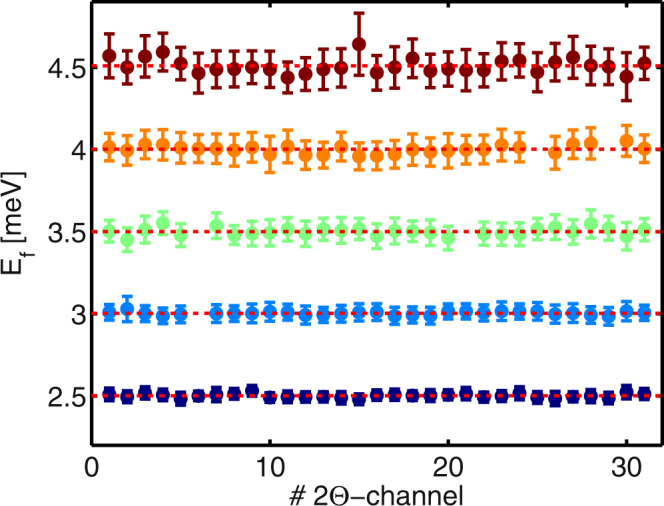
Results of the measurements of the elastic incoherent line in a vanadium sample (height 5 mm, diameter 5 mm). The peak energy of each channel is shown with the errorbar corresponding to the FWHM of the Gaussian fit. The different final energies are color-coded: $2.5\,$meV (dark blue), $3\,$meV (light blue), $3.5\,$meV (green), $4\,$meV (orange) and $4.5\,$meV (dark red). The mean final energy over all $2\theta$-channels with the same nominal final energy is shown by the red dotted line. The variations within corresponding ${E}_{f}$-channels are very small. 5 detectors are missing and the data points are absent in this plot.

In order to probe the inherent energy resolution of each channel of the MultiFLEXX backend, measurements of the elastic incoherent scattering signal from vanadium were performed. The incident energy ${E}_{i}$ was scanned in the range from $2.4$ to $4.875\,$meV. Since the energy resolution is affected by the sample height in the case of a vertically scattering analyzer geometry, the scans were repeated for vanadium heights of 5, 10, 15 and 30 mm (rod diameter 5 mm). The full-width-at-half-maximum (FWHM), peak and integrated intensity in energy of each channel were obtained from Gaussian fits to the elastic lines. Figure 4 shows the results for a vanadium height of 5 mm. Here, the fitted peak energy of each channel is plotted with the errorbar denoting the corresponding FWHM. The variations between the channels are very small. The alignment of each crystal set has been done optically by using the reflected beam of a laser. Here, the holder was rotated around the horizontal axis perpendicular to the scattered neutron beam such that the laser beam was focused onto the detector position. The results clearly demonstrate the high quality of the overall alignment.

**Figure 5:**
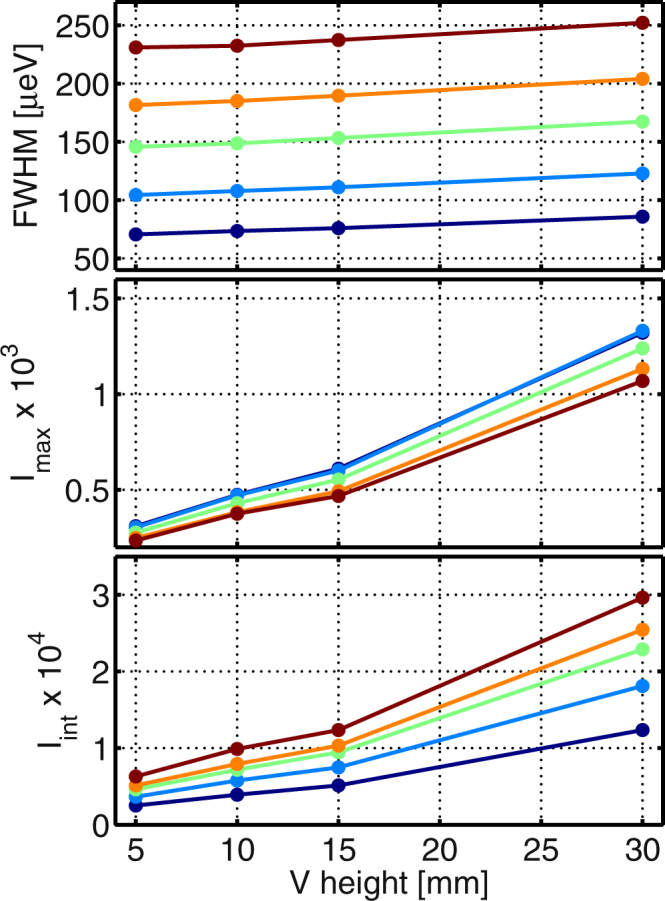
Mean values of the FWHM (top), peak intensity (middle) and integrated intensity (bottom) of the incoherent elastic line in vanadium as a function of sample height. The different ${E}_{f}$-channels are color-coded: $2.5\,$meV (dark blue), $3\,$meV (light blue), $3.5\,$meV (green), $4\,$meV (orange) and $4.5\,$meV (dark red).

The mean values of the FWHM, the peak intensity and the integrated intensity of the different nominal final energy channels are shown in Fig. 5 as a function of measured vanadium sample height. As seen in the top panel the sample height has only a small effect on the energy resolution. The obtained FWHM values are slightly larger than the linewidths expected at a classical TAS with similar final energies and a horizontal scattering geometry^25,30^. The peak intensity is larger for the lower ${E}_{f}$-channels, since these channels are closer to the sample and cover a larger solid angle. As already confirmed during the prototype measurements^32^ this effect is compensated for the integrated intensity due to the increase in resolution volume with increasing ${k}_{f}$ (${V}_{res}^{{E}_{f}}\propto {k}_{f}^{3}/tan({\theta }_{A})$). The intensities shown here have been normalized to monitor counts.

### Detector efficiency and intensity corrections

**Figure 6:**
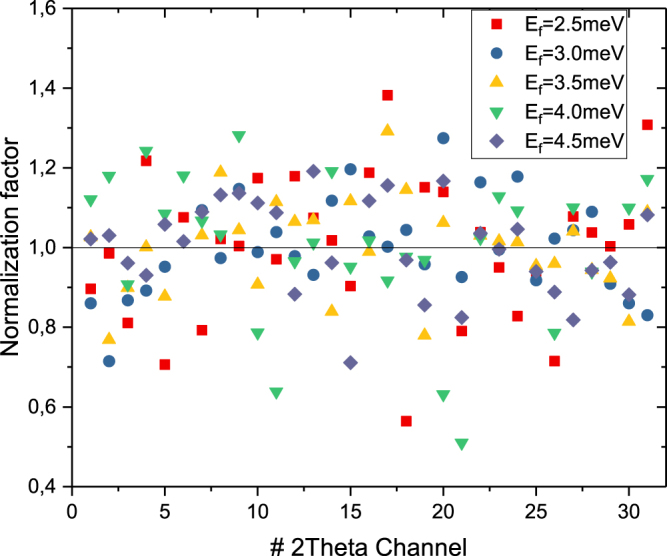
Normalization factor for each channel extracted from the measured vanadium incoherent line normalized to the average of all $2\theta$-channels of each energy channel.

Apart from the intensity correction due to the change in the resolution volume for the different final wavevectors, the intensity should also be corrected for the detector efficiency and the HOPG reflectivity. For the detector efficiency and overall intensity correction, a normalization factor has been extracted from the incident energy scans of the incoherent line of the vanadium sample. The reference for the normalization is the average intensity per energy channel of all the 31 $2\theta$-channels. Figure 6 shows the normalization factor extracted from the vanadium incoherent line for each detector. The normalization factors are homogeneously scattered around 1 without any major outliers. The variations are due to slight differences in analyzer reflectivity and detector efficiency.

### Comparison to standard FLEXX backend

The performance of the MultiFLEXX backend in regards of signal-to-noise-ratio was compared to the standard FLEXX backend using the elastic incoherent signal of vanadium. These measurements provide a reference point for determining the feasibility of an experiment on MultiFLEXX from preliminary FLEXX data.

A vanadium sample ($h=2\,$cm) was mounted within the VM-1B 15 T vertical magnet. The VTI of the magnet was set to $T=135\,$K and filled with exchange gas. The signal-to-noise-ratio was determined using ${E}_{f}=3.5\,$meV and incident energies of ${E}_{i}=3.5$ and 5 meV. For the standard FLEXX backend a ratio of 1441 (peak: 4852 counts/min, background 3.4 counts/min) was measured. For MultiFLEXX the average of the third analyzer channels (${E}_{f}=3.5\,$meV) was used and a ratio of 1655 was obtained. Thus the MultiFLEXX backend can easily compete with the standard FLEXX TAS backend in terms of signal-to-noise-ratio. The peak intensity for the standard FLEXX backend is a factor of 10 larger compared to MultiFLEXX, which is mainly determined by the increased analyzer area. However, this factor is by far exceeded by the large (${\bf{Q}},\omega$)-coverage of MultiFLEXX with $155$ channels which results in a total gain factor of $\approx 15$ in data collection efficiency. This estimated gain factor does not take differences in resolution into account, as such detailed comparison of the two backends is difficult for a number of reasons. For one thing, the final energy of any given channel on the MultiFLEXX is fixed, with a correspondingly fixed energy resolution. Conversely, the wavevector resolution using the MultiFLEXX backend is naturally very good, due to the collimation effect of the small analyzers. Using a collimator between the monochromator and sample thus greatly improves measurement efficiency with good wavevector resolution of the MultiFLEXX as compared to its standard TAS counterpart. Whether or not this altered flexibility of the resolution function is an advantage is very much dependent on the experiment in question. Therefore, we find that a simple comparison of covered spatial angle is the most transparent comparison of the two backends.

### Inelastic neutron scattering experiments

#### Magnetic excitations in MnF_2_

**Figure 7:**
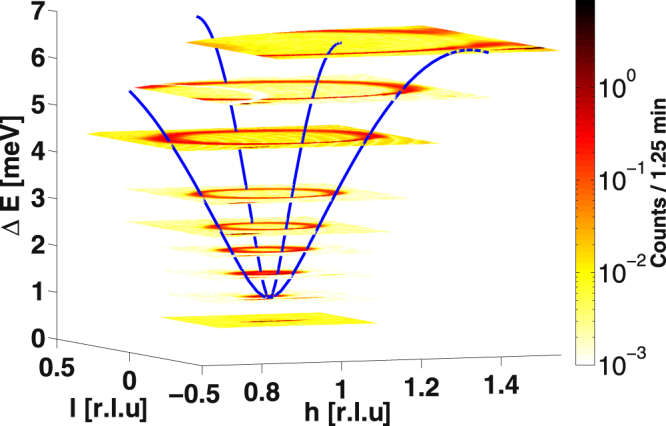
Constant energy cuts through the magnon dispersion of MnF_2_. The maps were measured using three different incident energies. With increasing energy transfer the evolution of the magnetic excitation out of the (1 0 0) Bragg peak is clearly visible. For clarity only every second map is shown. Blue lines represent the calculated dispersion along the high-symmetry directions.

In order to characterize the mapping capabilities of MultiFLEXX magnetic excitations of MnF_2_ were measured. MnF_2_ is a strong and well understood antiferromagnet with magnetic Mn^2+^ ions with $S=\mathrm{5/2}$ ^37,38^. The sample (a disk of $2\,$cm diameter and $0.5\,$cm height, $m=6.2\,$g) was aligned in the (H 0 L) plane and cooled to $T=14.78\,$K. The spin wave dispersion evolving from the (1 0 0) Bragg peak was mapped out in $27\,$hours with sample rotation scans. Using incident neutron energies of ${E}_{i}=5.1$, $5.8$ and $9\,$meV and corresponding energy transfers from ${\rm{\Delta }}E=0.6$ to $6.5\,$meV the entire dispersion was covered with a total of 15 constant energy maps. The results are shown in Fig. 7. For better clarity only every second map is shown. With increasing energy transfer a clear evolution of the magnon dispersion is visible. For comparison the calculated dispersion along high-symmetry directions defined by the coupling constants given in ref.^37^ is shown. The experimental data are in excellent agreement with the theoretical description.

Although the lowest energy transfer measured (${\rm{\Delta }}E=0.6\,$meV) is well below the spinwave gap of 1 meV, there is still some intensity recorded. This is a resolution effect as discussed by Toft-Petersen *et al*.^32^. The energy transfer is still close enough to the (1 0 0) antiferromagnetic Bragg peak so that the resolution ellipsoid catches some of the Bragg tail intensity producing localized intensity around (1 0–0.05). The single spurions present in slices corresponding to a higher energy transfer are also due to the strong (1 0 0) antiferromagnetic Bragg peak causing diffuse scattering background from the HOPG analyzer crystals^32^. A second resolution effect is the difference in peak width when scanning through the dispersion at high energy transfers. This is due to the changing slope of the resolution ellipsoid for different momentum transfers (focused and defocused to the slope of the dispersion).

**Figure 8:**
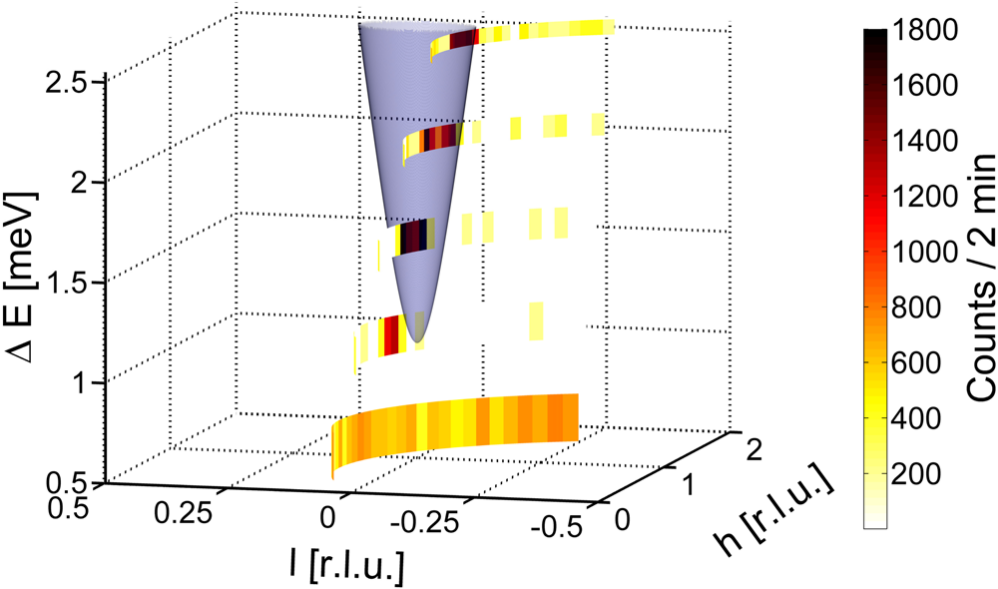
Constant energy lines recorded at the sample base temperature of $T=14.78\,$K. The constant energy lines cut through the magnon dispersion of MnF_2_ (semi-transparent).

While cooling the sample measurements were performed to demonstrate the capabilities of MultiFLEXX for parametric studies. The scattering and sample angle were fixed and scattered neutrons were repeatedly recorded for a counting time of 2 minutes. Thus, for a single scattering angle and sample angle MultiFLEXX records 5 constant energy lines as a function of sample temperature. As an example the results for the base temperature of $T=14.78\,$K are shown in Fig. 8. The dispersion cone according to the theoretical parameters is plotted as a semi-transparent surface. It is clearly visible where the constant energy lines cut the dispersion surface and the experimental data are in excellent agreement with the theoretical model. Here, a clear condensing of the paramagnetic scattering onto the spin wave dispersion below the ordering temperature can be seen.

#### Magnetic excitations in Ho

The magnetic excitations in the one-spin-slip phase of Ho were mapped out using MultiFLEXX. Ho has a very rich phase diagram, going through an antiferromagnetic helical phase between 130 and $18\,$K, and entering a ferromagnetic commensurate conical phase below temperatures of $18\,$K. In the helical phase, the magnetic moments are aligned ferromagnetically in the basal plane and the alignment direction rotates in successive planes. This magnetic order is characterized by an incommensurate wavevector, which is temperature-dependent. Magnetic Bragg peaks in this phase appear as satellites around structurally allowed and forbidden Bragg reflections^39,40^. The nature of the magnetic structure with rapidly changing periodicity makes the magnetic excitations in this compound unconventional. They show strong changes as a function of temperature. The one-spin-slip structure introduces gaps in the spin wave dispersion. The dispersion relation of these excitations along the crystallographic c* axis was investigated by McMorrow, *et al*.^41^. The data are well described by a random phase approximation model by Jensen^42^.

During the commissioning of MultiFLEXX, the whole dispersion relation of these excitations in the (0 K L) plane was measured. A Ho single crystal with a total mass of $1.75\,$g was aligned with (0 K L) in the scattering plane and cooled down to $19.5\,$K by a standard Orange cryostat. For this experiment the radial collimator was installed. The data were collected at three different nominal 2$\theta$ positions of the multi-analyzer setup to acquire an extensive region of the reciprocal space. For every 2$\theta$ position a 120° scan of the sample rotation angle ($A3$) has been performed with 1° steps and a monitor of 4000000 was accumulated in 240 seconds for every step. This resulted in a total experiment time of 24 hours.

**Figure 9:**
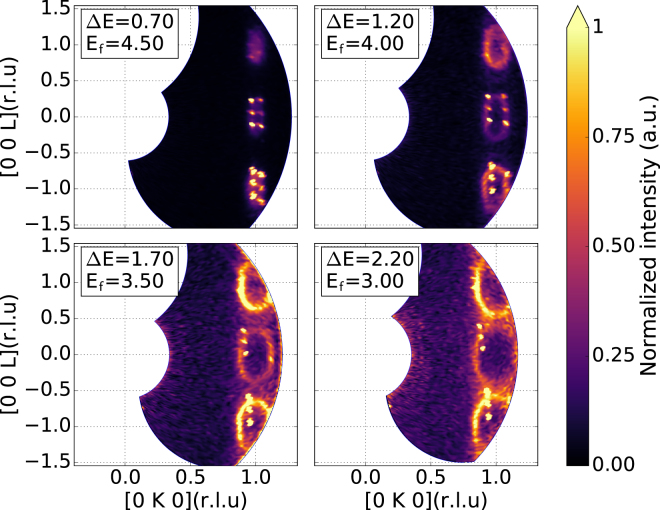
Constant energy maps of the dispersion relation of magnetic excitations in Ho as measured by MultiFLEXX. Oval rings show the intersection of the measurement plane with the slightly anisotropic dispersion surface. Bragg spurions are clearly evident and can be readily masked out.

Figure 9 shows four different constant energy maps of a large region of reciprocal space. There are two distinct features in those data: Oval-shaped magnetic excitations centered along the (0 1 L) direction and spurions arising from the magnetic Bragg peak and the two magnetic satellites. This results in 6 evident spurions which can be easily identified. The measured dispersion relation shown in Fig. 9 is in good agreement with the data published by McMorrow, *et al*.^41^. Contrary to standard TAS experiments the MultiFLEXX data probe the complete dispersion relation including off-symmetry directions in the (0 K L) scattering plane over several magnetic Brillouin zones. This gives direct access to structure factor variations as demonstrated by the varying intensity of the different dispersion cone intersections. This is a major advantage of measurements with the MultiFLEXX backend over a classic neutron triple-axis spectrometer.

#### Spurions

As on classical TAS spectrometers spurious signals may contaminate the inelastic neutron scattering signal of interest. On the one hand multiplexing techniques are more vulnerable to spurious signals, on the other hand the large $({\bf{Q}},\omega )$-coverage offers analysis strategies to identify these spurious signals readily. In the following we show that contaminating signals which arise from parasitic Bragg scattering with MultiFLEXX are easily identified by their wavevector-energy-relationship.

Neighboring $2\theta$-channels are well shielded by Cd sheets and the bulk BPE shielding material of each cassette amounting to a total of $2\,$mm Cd and 0 to 40 mm of BPE depending on the distance to the sample position. As a result, there is no measurable crosstalk between neighboring channels. However, strong Bragg peaks can cause spurious signals within a single $2\theta$-channel due to parasitic reflections of the analyzer crystals, which act like a graphite filter for higher incident energies. For investigating the effect of spurions a sample with strong elastic scattering and a weak inelastic signal near the elastic line was chosen. The sample used, PbCuTe_2_O_6_ (mosaicity 1.6°, total sample mass 1.3 g and dimensions $1.5\times 0.5\times 0.5\,$cm^3^), shows only a very weak diffuse inelastic signal. For this experiment the radial collimator was not available. The Bragg peaks for this sample are strong resulting in a count rate of 1000 counts/second at the $\mathrm{(2}\bar{2}\mathrm{0)}$ reflection.

**Figure 10:**
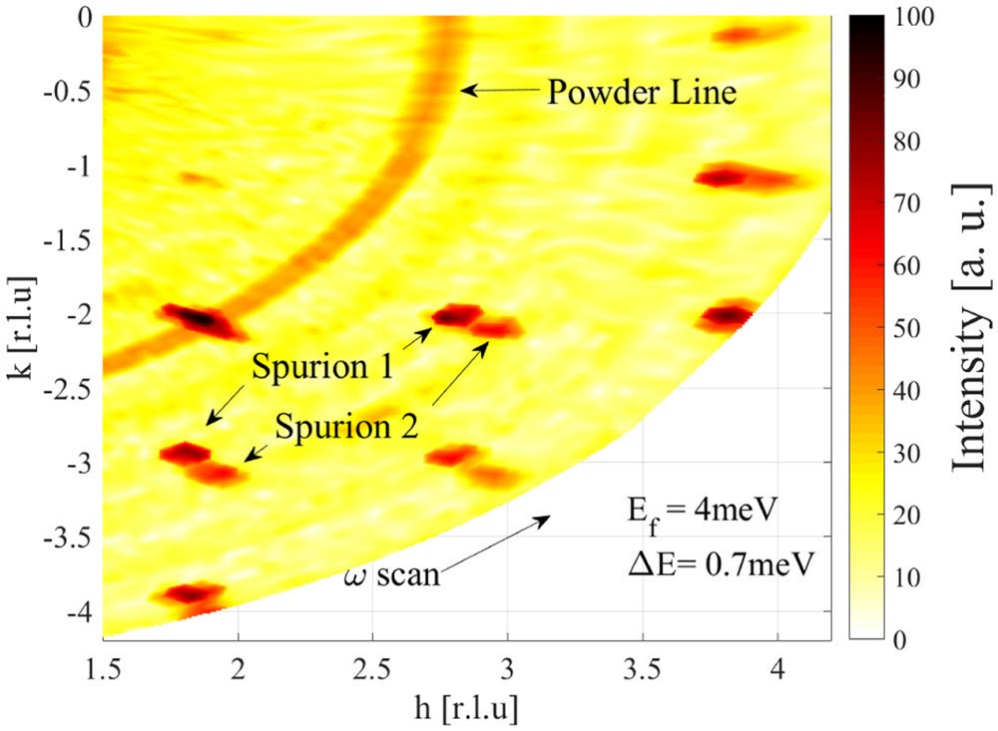
Constant energy map for PbCuTe_2_O_6_ at an energy transfer of 0.7 meV, taken with the penultimum energy channel E_*f*_ = 4 meV. The color scale represents neutron counts in 5 min.

Rocking scans were performed with an incident energy of ${E}_{i}=4.7\,$meV. Figure 10 shows the intensity distribution in the (H K 0) wavevector plane at an energy transfer of ${\rm{\Delta }}E=0.7\,$meV. Two different types of Bragg spurions are evident in the data. The first one (spurion 1) is a direct contamination from the Bragg peak through a diffuse scattering process which does not satisfy the elastic conditions. This kind of spurion is present only in a single $2\theta$-channel where it affects all energy channels. The second type of spurion (spurion 2) is due to a resolution effect. Here the resolution ellipsoid still overlaps with the tail intensity of the elastic signal resulting in a spurious inelastic signal. These spurions appear only in a single energy channel, however, at different $2\theta$-channels depending on the sample rotation angle $A3$.

**Figure 11:**
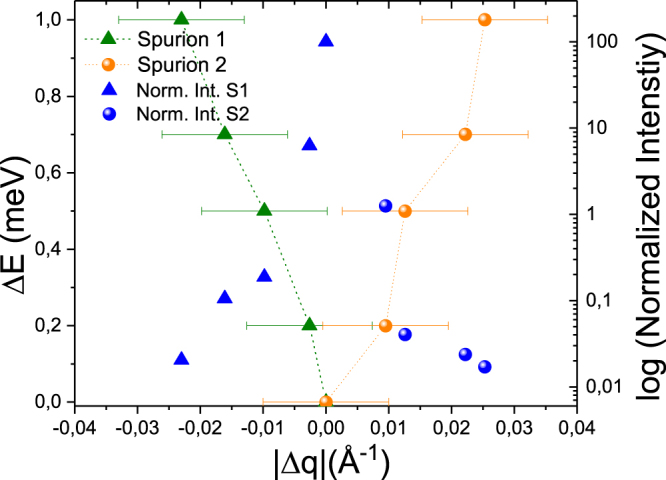
Wavevector-energy relation and intensity of the two types of Bragg spurions.

Figure 11 shows the wavevector-energy-relationships of the recorded spurions. Here, the modulus of the wavevector $|{\rm{\Delta }}{\bf{q}}|$ is defined as the difference between the total wavevector transfer and the corresponding Bragg peak. Both of these wavevector-energy-relationships are linear and the intensity of spurion 1 is larger than the intensity of spurion 2. Spurion 1 is present for all energy channels when the incident energy is less than $5.5\,$meV. For spurion 2 the slope of the wavevector-energy-relationship is related to the orientation of the resolution ellipsoid. The slope of the long axis of the resolution ellipsoid in the $({Q}_{\perp },\omega )$-plane close to the elastic condition is given by $\hslash {\rm{\Delta }}\omega /{\rm{\Delta }}{Q}_{\perp }\approx \mathrm{(4}\,$meVÅ^2^)*k* ^43^, which coincides with ${\rm{\Delta }}E/|{\rm{\Delta }}{\bf{q}}|$. The intensity of these two spurions decreases drastically with energy transfer resulting in less than 0.1% of the total Bragg peak intensity. However, this intensity gets relevant when comparing to typical magnetic inelastic intensities and typical counting times in real experiments.

Apart from the Bragg peak spurions the data show a powder line arising from the sample holder and/or sample environment. Powder lines are easily identified by their ring-like structure in the wavevector plane, since they do not depend on sample rotation angle $A3$. They can be masked out during analysis.

The spurious signals are typical for multiplexing instruments, which use HOPG analyzer crystals. The implemented velocity selector is key to remove higher-order neutrons and to minimize spurious effects. The remaining spurious effects can easily be identified and masked out. However, when signals are weak, background scans are advisable as it is currently the practice on standard time-of-flight machines. The effect of spurions could be further minimized by using an additional Be filter in front of MultiFLEXX.

#### Background

MultiFLEXX will be operated with the standard sample environment suite available at FLEXX (cryostats, furnaces, cryo-magnets, pressure cells). The single $2\theta$-channels have a rather large horizontal acceptance angle and in principle act like a coarse radial collimator. However, the acceptance angle of the different ${E}_{f}$-channels decreases with increasing distance from the sample. For the analyzer modules closer to the sample the acceptance angle is still large enough to record a significant incoherent background signal from the variable temperature insert (VTI) of the SE. Therefore, an additional radial collimator is installed (specification see section 2). In order to investigate the background signal arising from SE several ${E}_{i}$-scans were performed with a Variox cryostat and the VM-1B vertical 15 T split coil magnet.

Using the Variox cryostat in combination with the radial collimator the background decreases from 1 count/min to 0.5 count/min from ${E}_{f}$-channel 1 to 5 (${E}_{i}=7.5\,$meV) due to the increased amount of shielding given by the design of MultiFLEXX. The background for the detectors at high $2\theta$-channels is slightly higher due to the proximity to the monochromator shielding. For ${E}_{i}=5\,$meV, close to the nominal energy of the fifth analyzer segment, the background starts to increase (2.3 counts/min), since the resolution volume starts to overlap with the elastic incoherent line. For incident energies larger than 9 meV the background in the first channels (nominal energy 2.5 meV) increases drastically (up to 17 counts/min) due to higher-order Bragg scattering of the analyzer.

A similar background is observed for the VM-1B 15 T vertical magnet in combination with the radial collimator. The background for incident energies different from the nominal first order energy of the fifth analyzer and the second order energy of the first analyzer are even lower (0.6 count/min to 0.15 count/min from ${E}_{f}$-channel 1 to 5, ${E}_{i}=7.5\,$meV). A comparison to a setup without collimator shows, that the background of the single channels increases with decreasing distance to the sample, as expected from the geometry. The radial collimator reduces the background of the channels by a factor of 5, 3.1, 3, 2.4 and 1.3 for ${E}_{f}$-channels 1 to 5 (at an incident energy ${E}_{i}=7.5\,$meV).

In addition, to the existing sample environment suite an in-house redesign of an Orange cryostat with a very low and flat background is available. For this cryostat two important modifications of the VTI sample space have been implemented: The wall thickness of the VTI tube has been reduced to 0.2 mm in order to minimize the amount of material in the neutron beam. In addition, the diameter of the VTI sample tube has been significantly increased from 50 mm to 68 mm. In combination with the radial collimator the additional material in the neutron beam, which could scatter neutrons towards the analyzers, is further reduced. To guarantee a safe operation a copy of the thin walled VTI sample tube was tested to withstand pressures up to 11.5 bar before destruction.

## Conclusions

The newly built multi-analyzer module MultiFLEXX for the cold triple-axis spectrometer FLEXX has been characterized in terms of its mapping capabilities, energy resolution, detection efficiency and background. The setup was shown to be ideal to map out entire dispersion surfaces in a single scattering plane for a broad range of energy transfers. This was demonstrated with magnetic excitations in MnF_2_ and Ho single crystal samples. Typically, a complete data set with off-symmetry axis information on quasi-particle dynamics can be obtained in less than 4 days. The data collection efficiency compared to the standard TAS mode is significantly enhanced by a factor of 15. The energy resolution is only slightly more relaxed (by $\approx 60\, \%$ for our choice of the incident neutron energy) than the energy resolution on a standard TAS backend. The vertical spatial dimension of the sample was shown to have only a very weak influence on the energy resolution. Systematic variations in detected intensity such as solid angle coverage and volume of the resolution function are largely balanced out by the instrument design. This results in homogenous sensitivity of the individual detection channels and a small variation of the normalization factors. Two types of spurious signals have been identified. Their wavevector-energy characteristics have been shown to unambiguously identify their origin. This provides means for data analysis strategies which mask out this erroneous signals. Due to the successful shielding concept the MultiFLEXX backend is competitive with the standard FLEXX in terms of background. The MultiFLEXX backend was proven to be a powerful new tool for efficiently performing overview studies of low-energy dynamics.
